# Detecting Amyloid Positivity in Elderly With Increased Risk of Cognitive Decline

**DOI:** 10.3389/fnagi.2020.00228

**Published:** 2020-07-30

**Authors:** Timo Pekkala, Anette Hall, Tiia Ngandu, Mark van Gils, Seppo Helisalmi, Tuomo Hänninen, Nina Kemppainen, Yawu Liu, Jyrki Lötjönen, Teemu Paajanen, Juha O. Rinne, Hilkka Soininen, Miia Kivipelto, Alina Solomon

**Affiliations:** ^1^Institute of Clinical Medicine/Neurology, University of Eastern Finland, Kuopio, Finland; ^2^Public Health Promotion Unit, Finnish Institute for Health and Welfare, Helsinki, Finland; ^3^Division of Clinical Geriatrics, Center for Alzheimer Research, NVS, Karolinska Institutet, Stockholm, Sweden; ^4^VTT Technical Research Centre of Finland Ltd., Tampere, Finland; ^5^Neurocenter/Neurology, Kuopio University Hospital, Kuopio, Finland; ^6^Turku PET Centre, University of Turku, Turku, Finland; ^7^Division of Clinical Neurosciences, Turku University Hospital, Turku, Finland; ^8^Department of Clinical Radiology, Kuopio University Hospital, Kuopio, Finland; ^9^Combinostics, Tampere, Finland; ^10^Finnish Institute of Occupational Health, Helsinki, Finland; ^11^Institute of Public Health and Clinical Nutrition, University of Eastern Finland, Kuopio, Finland; ^12^Ageing Epidemiology Research Unit, School of Public Health, Imperial College London, London, United Kingdom

**Keywords:** amyloid beta, positron emission tomography, cognition, magnetic resonance imaging, apolipoprotein E, machine learning, Alzheimer’s disease

## Abstract

The importance of early interventions in Alzheimer’s disease (AD) emphasizes the need to accurately and efficiently identify at-risk individuals. Although many dementia prediction models have been developed, there are fewer studies focusing on detection of brain pathology. We developed a model for identification of amyloid-PET positivity using data on demographics, vascular factors, cognition, *APOE* genotype, and structural MRI, including regional brain volumes, cortical thickness and a visual medial temporal lobe atrophy (MTA) rating. We also analyzed the relative importance of different factors when added to the overall model. The model used baseline data from the Finnish Geriatric Intervention Study to Prevent Cognitive Impairment and Disability (FINGER) exploratory PET sub-study. Participants were at risk for dementia, but without dementia or cognitive impairment. Their mean age was 71 years. Participants underwent a brain 3T MRI and PiB-PET imaging. PiB images were visually determined as positive or negative. Cognition was measured using a modified version of the Neuropsychological Test Battery. Body mass index (BMI) and hypertension were used as cardiovascular risk factors in the model. Demographic factors included age, gender and years of education. The model was built using the Disease State Index (DSI) machine learning algorithm. Of the 48 participants, 20 (42%) were rated as Aβ positive. Compared with the Aβ negative group, the Aβ positive group had a higher proportion of *APOE* ε4 carriers (53 vs. 14%), lower executive functioning, lower brain volumes, and higher visual MTA rating. AUC [95% CI] for the complete model was 0.78 [0.65–0.91]. MRI was the most effective factor, especially brain volumes and visual MTA rating but not cortical thickness. *APOE* was nearly as effective as MRI in improving detection of amyloid positivity. The model with the best performance (AUC 0.82 [0.71–0.93]) was achieved by combining *APOE* and MRI. Our findings suggest that combining demographic data, vascular risk factors, cognitive performance, *APOE* genotype, and brain MRI measures can help identify Aβ positivity. Detecting amyloid positivity could reduce invasive and costly assessments during the screening process in clinical trials.

## Introduction

The importance of dementia prevention and early interventions in Alzheimer’s disease (AD) (Winblad et al., 2016) has emphasized the increasing need for accurate identification of at-risk individuals who may benefit most from such interventions. Although many dementia prediction models have been developed (Hou et al., 2019), there are considerably fewer studies focusing on detection of brain pathology. Given the central role attributed to beta-amyloid (Aβ) pathology in AD (Dubois et al., 2007), early identification of individuals with Aβ pathology has become particularly important.

The prevalence of Aβ pathology from ages 50 to 80 years has been estimated to range from 10 to 33% in cognitively normal individuals, and from 27 to 60% in individuals with mild cognitive impairment (MCI) (Jansen et al., 2015). This complicates the screening process in e.g., randomized controlled trials testing interventions that target Aβ, since assessment of Aβ pathology in cerebrospinal fluid (CSF) or on positron emission tomography (PET) scans can easily become inefficient due to invasiveness, costs, and/or PET availability. Developing models for detecting Aβ pathology based on less invasive, less costly, and more easily available factors could help identify a target population with high prevalence of Aβ pathology. More selective use of CSF or PET assessments to confirm the presence of Aβ pathology could thus reduce costly screening failures and improve screening efficiency.

Previous models for Aβ pathology, were most commonly developed in mixed populations including individuals with AD dementia and/or MCI (e.g., Bahar-Fuchs et al., 2013; Tosun et al., 2013, 2014; Burnham et al., 2014; Apostolova et al., 2015; Haghighi et al., 2015; Lee et al., 2018; Westwood et al., 2018; Palmqvist et al., 2019; Ansart et al., 2020), with area under the receiver operating characteristic curve (AUC) values up to 0.87–0.88. Very few studies have focused specifically on cognitively normal populations, despite the key importance of this group who could potentially benefit from interventions that are started early, before the onset of cognitive impairment. Lower performance has been reported for models developed in cognitively normal individuals, with AUC values up to 0.74–0.77 (Mielke et al., 2012; Insel et al., 2016; ten Kate et al., 2018; Ansart et al., 2020). Models for detecting Aβ pathology have most often been developed based on demographic data, cognitive performance, and apolipoprotein E (*APOE*) genotype. Of the studies in cognitively normal individuals, two have also included structural magnetic resonance imaging (MRI) (ten Kate et al., 2018; Ansart et al., 2020).

In this study, we first aim to develop a model for detecting Aβ pathology in individuals with risk factors for dementia, but without dementia or substantial cognitive impairment. We assess a broad range of factors, including demographic data, cardiovascular factors, cognitive performance, *APOE* genotype, and brain MRI measures. Both visual rating of medial temporal lobe atrophy (MTA) and quantitative measures of regional brain volumes and cortical thickness are considered. The second aim is to conduct a pragmatic analysis on the added value of the different factors, taking into account how easily obtainable they are in clinical settings, i.e., from less complex to more specialized. The model uses baseline data from the Finnish Geriatric Intervention Study to Prevent Cognitive Impairment and Disability (FINGER) exploratory PET sub-study.

## Materials and Methods

### Participants

The FINGER main study design and population characteristics have been previously described (Kivipelto et al., 2013; Ngandu et al., 2014). In brief, FINGER (ClinicalTrials.gov identifier NCT01041989) was a 2-year randomized controlled trial testing a multidomain lifestyle intervention versus regular health advice in 1260 older individuals at risk for dementia from the general population. Results showing intervention benefits on the primary and secondary cognitive outcomes, as well as on several other outcomes, have been published (Ngandu et al., 2015).

The exploratory PET sub-study included 48 individuals from the Turku site in FINGER main study and was conducted at the Turku PET Centre. The 48 participants were selected from the most recently recruited individuals when MRI/PET resources became available at the Turku site, and if there were no contraindications. Participants had to be eligible for MRI and PET scans, in addition to meeting all inclusion criteria for the FINGER main study: age 60 to 77 years; Cardiovascular Risk Factors, Aging, and Dementia (CAIDE) score at or above six points indicating elevated risk for dementia; and cognitive performance at the mean level or slightly lower than expected for age according to Finnish population norms for the Consortium to Establish a Registry for Alzheimer’s Disease (CERAD) test as previously described in detail (Kivipelto et al., 2013). Participants had to meet at least one of the following criteria: word list memory task results ≤19 words; word list recall ≤ 75%; or mini mental state examination ≤26 points. Individuals with diagnosed dementia or suspected dementia or substantial cognitive impairment based on screening assessments were excluded from the study. Additionally, participants with MMSE score < 20, a disorder that would affect safe engagement in the intervention, loss of hearing, vision or ability to communicate, a disorder preventing cooperation; or participation in another intervention trial were also excluded.

The FINGER PET population was not significantly different from the rest of the Turku cohort or the rest of the FINGER participants regarding education, vascular risk factors, or *APOE* e4 carrier status (Kemppainen et al., 2018). FINGER PET participants were slightly older than the rest of the FINGER population at the baseline visit (mean 70.8 vs. 69.3 years), most likely due to a later initiation of recruitment at the Turku study site (Kemppainen et al., 2018).

The FINGER study was approved by the Coordinating Ethics Committee of the Hospital District of Helsinki and Uusimaa. All participants gave written informed consent at the screening and baseline visits, and also for the exploratory neuroimaging sub-study.

### Clinical Assessments and APOE Genotyping

The present study used data from the FINGER baseline visit, before the start of the intervention. Cognition was measured using a modified version of the Neuropsychological Test Battery (mNTB) (Harrison et al., 2007). A standardized composite mNTB score was determined based on 14 individual tests measuring three different cognitive domains, i.e., memory, executive function, and processing speed. Domain-specific standardized mNTB scores were also calculated as previously described (Ngandu et al., 2015), with higher scores indicating better performance. Height, weight, and blood pressure were measured (Ngandu et al., 2014), and body mass index (BMI) and hypertension (systolic blood pressure ≥140 mmHg and/or diastolic blood pressure ≥90 mmHg) were used as vascular risk factors in the model. Genomic DNA was extracted from venous blood samples with Chemagic MSM1 (PerkinElmer) using magnetic beads. *APOE* genotype was determined by polymerase chain reaction using TaqMan genotyping assays (Applied Biosystems) for two single-nucleotide polymorphisms (rs429358 and rs7412) and an allelic discrimination method on the Applied Biosystems 7500 platform (De La Vega et al., 2005).

### MRI and PET Imaging

Participants in the FINGER PET sub-study underwent a brain 3T MRI (Philips Ingenuity TF PET/MR, Amsterdam, Netherlands) and ^11^C-Pittsburgh compound B (PiB)-PET imaging. The MRI and PiB-PET protocols have been previously published (Kemppainen et al., 2018). PiB images were visually determined as positive or negative by two-party consensus agreement. PiB negative individuals had only non-specific ^11^C-PiB-PET retention in white matter, whereas PiB positive individuals had ^11^C-PiB-PET retention in at least one AD-specific cortical region.

Regional brain volumes and cortical thickness were measured on MRI scans using FreeSurfer (version 5.3)^1^. Brain volumes were normalized to total intracranial volume to take into account variations in head size. An AD-signature cortical thickness measure was calculated as the average of cortical thickness in the entorhinal, inferior and middle temporal, and fusiform regions (Jack et al., 2015). Additionally, visual assessment of MTA was conducted by a single rater blinded to clinical data based on a T1-weighted coronal slice. MTA was graded on the Scheltens scale from 0 to 4 (Scheltens et al., 1992).

### Statistical Analysis

The population was characterized by calculating group means and standard deviations. Statistical significance of group differences was examined using the Wilcoxon rank sum test for continuous and categorical data.

We used a machine-learning algorithm (Disease State Index, DSI) to detect PiB-PET positivity with clinical, *APOE* and MRI data as factors. DSI is a supervised machine learning method developed at the VTT Technical Research Centre of Finland (Mattila et al., 2011). Its accuracy is comparable to other methods such as logistic regression, support vector machines, and Bayes inference (Mattila et al., 2011), and it has been successful in modeling MCI progression (Hall et al., 2015) and discriminating between dementia types (Koikkalainen et al., 2016; Tolonen et al., 2018). DSI classifies individuals into Aβ positive and negative based on a population with known Aβ status (training population). An individual’s data are compared with value distributions in the training population with a fitness function.

(1) $$f\,\left(x\right)=\frac{F\,N\,\left(x\right)}{F\,N\,\left(x\right)+F\,P\,\left(x\right)}$$

The fitness function *f* calculates a value for each measurement value *x*, where *FN* is the false negative error and *FP* is the false positive error for prediction at measurement value *x*. The analysis puts more weight on factors that show more pronounced dissimilarities between the positive and negative groups in the training population with a relevance value. The relevance is calculated by adding sensitivity and specificity and substracting 1.

(2) r⁢e⁢l⁢e⁢v⁢a⁢n⁢c⁢e=s⁢e⁢n⁢s⁢i⁢t⁢i⁢v⁢i⁢t⁢y+s⁢p⁢e⁢c⁢i⁢f⁢i⁢c⁢i⁢t⁢y-1.

This is also known as the Youden index. The grouped index values, such as overall MRI, are calculated by taking a relevance weighted average over fitness of each measurement. Recursively, a new fitness and relevance is defined for groups, from which the total DSI value is defined.

(3) $$D\,S\,I=\frac{\sum_{i}r\,e\,l\,e\,v\,a\,n\,c\,e_{i}\times f\,i\,t\,n\,e\,s\,s_{i}}{\sum_{i}r\,e\,l\,e\,v\,a\,n\,c\,e_{i}}$$

The resulting DSI value for an individual represents a point on the scale 0–1, where higher values denote higher similarity to Aβ positive individuals in the training population. A separate training population was not used in this study, but the data were cross-validated by randomly selecting 80% of the population for training and 20% for testing, and then repeating the procedure 100 times for statistically reliable results (100 × 5-fold cross validation). The classification results are shown as area under the receiver-operator curve (AUC) for the model, with 95% confidence intervals (CI) averaged from the folds.

Advantages of the DSI include the ability to incorporate a large number of factors simultaneously, and permissive requirements for types and distribution characteristics of the data. Missing values are ignored as part of the model, and the total DSI value is calculated from the available data. Over learning is a challenge especially when the sample size is small. DSI defines a classifier for each predictor separately and combines these classifiers making it less sensitive to over learning than methods based on complex decision boundaries. Conceptually related and potentially correlated factors can be structured into groups to assess their combined effect. Individual factors are combined into a group DSI value through a weighted average, and the process is then repeated recursively for all groups to obtain a total DSI value. DSI thus provides detailed information about performance on multiple levels simultaneously: the independent performance of each factor, the combined performance of a group of similar factors, and the overall performance of the entire model.

In this study, factors were organized into groups according to conceptual likeness: Demographic (age, sex, education), Cardiovascular (BMI, hypertension), *APOE* genotype (ε4 carrier vs. non-carrier), Cognition (mNTB total, memory, processing speed, and executive function), and MRI. Subgroups were defined for MRI measures (Volumes, Visual MTA score, and AD-signature cortical thickness) for a more detailed assessment of performance. Additional analyses were conducted to assess the added value of different factor groups (modalities), taking into account how easily obtainable they are in clinical settings. This was done by assessing performance of the model after step-by-step inclusion or exclusion of different factor groups.

All analyses were performed using MATLAB R2015b. DSI values were calculated using Fingerprint Toolbox version 0.9 on MATLAB.

## Results

Population characteristics according to Aβ status on PiB-PET scans are shown in Table 1. Of the 48 participants, 20 (42%) were rated as Aβ positive. Compared with the Aβ negative group, the Aβ positive group had a higher proportion of *APOE* ε4 carriers (53 vs. 14%), lower executive functioning, lower brain volumes (total cortical and gray matter volumes, cerebellar cortex, thalamus proper, putamen, hippocampus, amygdala, the accumbens area, and ventral diencephalon), and higher visual MTA rating. No significant differences were found in other characteristics (Table 1).

**TABLE 1:** Population characteristics according to amyloid status on PiB-PET scans.

	Mean (SD)		
	
	Amyloid- (*n* = 28)	Amyloid + (*n* = 20)	*p*-value
**Demographic**			
Sex/Female	14 (50%)	8 (40%)	0.505
Age (years)	70.2 (5.8)	71.6 (3.5)	0.310
Education (years)	9.7 (2.9)	8.9 (2.0)	0.320
**Cardiovascular**			
Body mass index	27.9 (3.6)	26.2 (2.6)	0.088
High blood pressure	10 (36%)	9 (45%)	0.529
***APOE*** (e4 carrier)^†^	4 (14%)	10 (53%)	0.005
**Cognition**			
mNTB Total	0.04 (0.53)	−0.09 (0.50)	0.421
mNTB Memory	−0.11 (0.52)	0.04 (0.64)	0.385
mNTB Processing speed	0.16 (0.95)	−0.10 (0.77)	0.184
mNTB Executive function	0.16 (0.58)	−0.22 (0.44)	0.026
**MRI**			
**Volumes**			
Total cortex	0.29 (0.03)	0.27 (0.03)	0.007
Total gray matter	0.39 (0.05)	0.36 (0.04)	0.009
Cerebellum cortex	0.063 (0.009)	0.059 (0.008)	0.027
Thalamus proper	9.3×10^−3^ (1.2×10^−3^)	8.4×10^−3^ (1.1×10^−3^)	0.022
Caudate	4.9×10^−3^ (8.6×10^−4^)	4.5×10^−3^ (7.7×10^−4^)	0.070
Putamen	7.0×10^−3^ (1.3×10^−3^)	6.1×10^−3^ (1.0×10^−3^)	0.014
Pallidum	1.9×10^−3^ (3.5×10^−4^)	1.8×10^−3^ (2.9×10^−4^)	0.198
Brain Stem	0.014 (0.002)	0.014 (0.002)	0.229
Hippocampus	5.2×10^−3^ (9.7×10^−4^)	4.6×10^−3^ (8.1×10^−4^)	0.019
Amygdala	2.3×10^−3^ (4.6×10^−4^)	2.0×10^−3^ (3.0×10^−4^)	0.030
Accumbens area	6.6×10^−4^ (1.3×10^−4^)	5.6×10^−4^ (1.3×10^−4^)	0.004
Ventral diencephalon	5.0×10^−3^ (5.7×10^−4^)	4.7×10^−3^ (4.1×10^−4^)	0.037
Cerebrospinal fluid	8.8×10^−4^ (1.4×10^−4^)	8.1×10^−4^ (1.4×10^−4^)	0.171
Optic chiasm	1.4×10^−4^ (3.6×10^−5^)	1.2×10^−4^ (4.6×10^−5^)	0.164
Total corpus callosum	2.0×10^−3^ (4.3×10^−4^)	1.7×10^−3^ (3.8×10^−4^)	0.058
**Visual MTA rating** (Scheltens)	1.0 (0.7)	1.6 (0.7)	0.007
**AD-signature cortical thickness**	2.8 (0.1)	2.7 (0.1)	0.084

*The Wilcoxon rank sum test was used to calculate p-values for differences between amyloid positive and negative groups. *APOE*, Apolipoprotein E; mNTB, modified Neuropsychological Test Battery; MRI, magnetic resonance imaging; MTA, medial temporal lobe atrophy; AD, Alzheimer’s disease. †: Amyloid + *n* = 19.*

The performance of the complete DSI model, factor groups and individual factors is shown in Table 2. The AUC of the complete model after cross-validation was 0.78 (95% CI 0.65–0.91). Model AUC without cross-validation (training and testing with all individuals) was 0.88. Table 3 shows sensitivity, specificity, and positive and negative predictive values for different DSI cutoff values. For example, setting the DSI cutoff value for positive classification at 0.5 would identify a sub-population with a true Aβ positivity prevalence (positive predictive value, PPV) of 65%, with 69% sensitivity, 69% specificity, and 77% negative predictive value (NPV). If only individuals with a DSI value ≥ 0.5 undergo PiB-PET scans, this would lead to an increase in the rate of Aβ positive scans from 42% (observed in FINGER PET study participants) to 65%. Using a higher cutoff for Aβ positivity prediction, such as 0.6, could increase the positive scan rate to 74%, but at a lower sensitivity (39%). Similarly, using a lower cutoff, such as 0.4, the positive scan rate would be 57% and sensitivity 87%.

**TABLE 2:** Performance for the full model, factor groups and individual factors for detecting amyloid positivity.

	Amyloid + relative to amyloid-	AUC (95% CI)
**Composite DSI**		0.78 (0.65–0.91)
**Demographic**		0.54 (0.37–0.70)
Sex/Female	↓	0.48 (0.35–0.60)
Age	↑	0.45 (0.28–0.61)
Education (years)	↓	0.59 (0.43–0.75)
**Cardiovascular**		0.60 (0.46–0.75)
Body mass index	↓	0.65 (0.50–0.79)
High blood pressure	↑	0.49 (0.37–0.61)
***APOE*** (e4 carrier)	↑	0.69 (0.56–0.82)
**Cognition**		0.65 (0.49–0.81)
mNTB Total	↓	0.55 (0.38–0.72)
mNTB Memory	↑	0.54 (0.38–0.70)
mNTB Processing speed	↓	0.57 (0.41–0.73)
mNTB Executive function	↓	0.69 (0.53–0.84)
**MRI**		0.75 (0.61–0.89)
**Volumes**		0.72 (0.57–0.88)
Total cortex	↓	0.73 (0.59–0.88)
Total gray matter	↓	0.72 (0.57–0.88)
Cerebellum cortex	↓	0.69 (0.54–0.84)
Thalamus proper	↓	0.70 (0.55–0.85)
Caudate	↓	0.65 (0.49–0.81)
Putamen	↓	0.71 (0.56–0.87)
Pallidum	↓	0.61 (0.45–0.77)
Brain Stem	↓	0.61 (0.45–0.77)
Hippocampus	↓	0.70 (0.54–0.86)
Amygdala	↓	0.69 (0.53–0.85)
Accumbens area	↓	0.75 (0.62–0.89)
Ventral diencephalon	↓	0.68 (0.53–0.83)
Cerebrospinal fluid	↓	0.61 (0.44–0.78)
Optic chiasm	↓	0.60 (0.41–0.78)
Total corpus callosum	↓	0.62 (0.45–0.79)
**Visual MTA rating** (Scheltens)	↑	0.71 (0.59–0.84)
**AD-signature cortical thickness**	↓	0.65 (0.48–0.82)

*Upward arrow indicates the amyloid + group having a larger mean value. AUC values (95% confidence interval) are from 100 × 5-cross-validation. AUC, area under the receiver operating characteristic curve; DSI, disease state index; *APOE*, Apolipoprotein E; mNTB, modified neuropsychological test battery; MRI, magnetic resonance imaging; MTA, medial temporal lobe atrophy; AD, Alzheimer’s disease.*

**TABLE 3:** Classifier statistics for selected DSI cutoff levels for detecting amyloid positivity.

DSI cutoff	Sensitivity (%)	Specificity (%)	PPV (%)	NPV (%)	RPP (%)
0.0	100	0	42 (42–42)		100
0.1	100	1 (0–1)	42 (42–42)	100	100 (99–100)
0.2	100	11 (10–12)	45 (45–45)	100	93 (93–94)
0.3	99 (98–99)	26 (24–27)	49 (49–50)	98 (96–99)	85 (84–86)
0.4	87 (86–89)	49 (47–50)	57 (55–58)	87 (85–88)	66 (65–68)
0.5	69 (67–71)	69 (68–71)	65 (63–66)	77 (76–79)	47 (45–48)
0.6	39 (37–41)	89 (88–90)	74 (71–77)	68 (67–69)	23 (22–24)
0.7	17 (16–19)	96 (95–97)	77 (74–81)	62 (62–63)	9 (9–10)
0.8	12 (11–13)	99 (99–100)	94 (92–97)	61 (61–62)	5 (5–6)
0.9	1 (1–2)	100 (100–100)	100 (100–100)	59 (58–59)	1 (0–1)
1.0	0	100		58 (58–58)	0

*Values are mean values (95% confidence interval) from 100 × 5-cross-validation. Values with no confidence interval are exact values. DSI, Disease state index; PPV, positive predictive value; NPV, negative predictive value, RPP rate of positive prediction.*

Among the groups of factors included in the model, structural MRI measures together had the best performance, with an overall AUC (95% CI) of 0.75 (0.61–0.89). Within the MRI group, the most effective subgroups were volumetric measures (AUC 0.72, CI 0.57–0.88) and visual MTA rating (AUC 0.71, CI 0.59–0.84), while AD-signature cortical thickness had lower performance (AUC 0.65, CI 0.48–0.82). *APOE* ε4 carrier status had an AUC (95% CI) of 0.69 (0.56–0.82). The Cognition group of factors had an AUC (95% CI) of 0.65 (0.49–0.81), and within this group executive functioning had an AUC of 0.69 (0.53–0.84). Other cognitive measures did not have detection power. BMI was the strongest factor (AUC 0.65, CI 0.50–0.79) in the cardiovascular group, although the group level AUC (95% CI) was low at 0.60 (0.46–0.75). Demographic factors had the poorest performance with years of education having the highest within-group AUC of 0.59 (0.43–0.75), and age and sex showing no effect.

Table 4 shows the added value of different groups of factors in terms of model performance. In the first scenario, a base model with only demographic and cardiovascular data (AUC 0.56, CI 0.41–0.72) was augmented by adding a single factor group (Cognition, *APOE*, Visual MTA rating, or all MRI measurements). The AUC improved to 0.62–0.71, but only addition of *APOE* or MRI data led to a CI above 0.50 indicating a significant model. All MRI measurements together had the highest added performance (AUC 0.71, 95% CI 0.56–0.85), followed by *APOE* ε4 carrier status (AUC 0.69, CI 0.56–0.83).

**TABLE 4:** Added value of different groups of factors in terms of model performance.

		AUC (95% CI)
**Demographic and Cardiovascular**		0.56 (0.41–0.72)
and additionally	Cognition	0.62 (0.46–0.77)
	*APOE* (e4 carrier)	0.69 (0.56–0.83)
	Visual MTA rating	0.66 (0.51–0.81)
	MRI all modalities	0.71 (0.56–0.85)
**Demographic, Cardiovascular, and Cognition**		0.62 (0.46–0.77)
and additionally	*APOE*	0.71 (0.56–0.85)
	Visual MTA rating	0.66 (0.51–0.82)
	MRI all modalities	0.72 (0.58–0.87)
**Demographic, Cardiovascular, Cognition, and *APOE***		0.71 (0.56–0.85)
and additionally	Visual MTA rating	0.75 (0.62–0.89)
	MRI all modalities (complete model)	0.78 (0.65–0.91)
***APOE***		0.69 (0.57–0.81)
and additionally	Visual MTA rating	0.81 (0.69–0.92)
	MRI all modalities	0.82 (0.71–0.93)
**MRI subgroup analysis**		
**All MRI modalities**		0.76 (0.62–0.90)
without	Volumes	0.72 (0.57–0.86)
	Visual MTA rating	0.74 (0.60–0.89)
	AD-signature cortical thickness	0.76 (0.62–0.89)
**All MRI modalities and *APOE***		0.82 (0.71–0.93)
without	Volumes	0.79 (0.66–0.91)
	Visual MTA rating	0.82 (0.71–0.93)
	AD-signature cortical thickness	0.83 (0.71–0.95)

*In each scenario (highlighted in bold type), factor groups are added one-by-one to a base model. In the MRI subgroup analysis, factor groups are removed one-by-one from a base model. AUC values (95% confidence interval) are 100 × 5-cross-validated. MRI, magnetic resonance imaging; AUC, area under the receiver operating characteristic curve; *APOE*, Apolipoprotein E; MTA, medial temporal lobe atrophy; AD, Alzheimer’s disease.*

The second scenario used a base model including demographic and cardiovascular factors, and cognition (AUC 0.62, 95% CI 0.46–0.77). Adding *APOE* or all MRI measures enhanced the model performance to an AUC (95% CI) of 0.71 (0.56–0.85) and 0.72 (0.58–0.87), respectively. Visual MTA rating improved the AUC (95% CI) only to 0.66 (0.51–0.82).

The third scenario tested the added value of the simple visual MTA rating instead of the more comprehensive automated MRI measures. The base model using all factors except MRI led to AUC (95% CI) 0.71 (0.56–0.85). Visual MTA rating increased AUC to 0.75 (0.62–0.89), with the complete model performing at 0.78 (0.65–0.91). We also tested a fourth scenario focusing on the combination of *APOE* and MRI, the two best performing factor groups. *APOE* together with either visual MTA rating (AUC 0.81, 95% CI 0.69–0.92) or all MRI measurements (AUC 0.82, 95% CI 0.71–0.93) performed better as a combination than the complete model.

The relative importance of the different subgroups of MRI factors is shown in Table 4. MRI factor subgroups were removed one-by-one from a base model. With a base model including all MRI measures, AUC (95% CI) decreased from 0.76 (0.62–0.90) to 0.72 (0.57–0.86) by removing volumetric measures, and to 0.74 (0.60–0.89) by removing visual MTA rating. Removing AD-signature cortical thickness did not affect the performance of the base model. With a base model including all MRI measures and *APOE*, removing AD-signature cortical thickness slightly improved the model performance, while removing volumetric measures decreased the AUC.

## Discussion

Findings from the exploratory FINGER PET sub-study suggest that a model combining demographic data, vascular risk factors, cognitive performance, *APOE* genotype, and brain MRI measures can detect Aβ positivity in older at-risk individuals without dementia or substantial cognitive impairment. Given the lower prevalence of Aβ pathology among cognitively normal individuals (Jansen et al., 2015), such a model would facilitate the identification of populations with a considerably higher prevalence, thus reducing the number of invasive, time-consuming and costly assessments during e.g., the screening process in clinical trials. The DSI model allows selecting a suitable threshold depending on the situation. An index threshold of 0.5 gives the best balance with sensitivity and specificity of the model. Using a low cut-off, such as 0.4 would mean very high sensitivity, but a large number of subsequent PET scans. This would mostly remove the cases that are likely to be amyloid negative. Usually, when trying to find participants with amyloid pathology, it might be more useful to choose a higher cut-off, such as 0.6, to ensure that a large number of participants are not needlessly subjected to a PET scan to verify amyloid positivity. This could be useful in a scenario, where we needed to select study participants who were amyloid positive, but did not want to do a PET scan on the whole cohort due to cost issues or minimizing possible harm or inconvenience on the participants. Performance of the complete DSI model including MRI was AUC 0.78, and 0.71 without MRI. Both could be considered “acceptable” as per Hosmer et al. (2013) criteria. Previously reported models in cognitively normal individuals have had AUCs in the range of 0.60–0.74 (Mielke et al., 2012; ten Kate et al., 2018; Ansart et al., 2020), with the highest performance for a support vector machine (SVM) model combining demographics, cognitive performance, *APOE* genotype and detailed structural MRI measures (ten Kate et al., 2018).

Similar to the abovementioned SVM model (ten Kate et al., 2018), MRI and *APOE* were the best factors in this study. Brain volumes with the highest performance (AUC ≥ 0.70) were total cortical and gray matter volumes, and hippocampus, accumbens, thalamus and putamen volumes, which have been previously reported to be lower in cognitively normal Aβ positive individuals (ten Kate et al., 2018). Visual MTA rating was almost as effective as brain volumes, although it was not selected in the previous SVM model (ten Kate et al., 2018). The AD-signature cortical thickness had lower performance than brain volumes or visual MTA rating in the FINGER PET population.

Very few studies in cognitively normal participants have investigated the added value of structural MRI in the detection of Aβ pathology. One study reported that best results were obtained without MRI, and that change in cognition over time was a superior substitute to MRI in a multimodal prediction model (Ansart et al., 2020). In another study, MRI measures did have an added value above other factors (ten Kate et al., 2018). Similar findings emphasizing the added value of MRI were observed in the FINGER PET sub-study. In addition, the leave-one-out analysis of the MRI factor group indicated brain volumes as the best factors, while AD-signature cortical thickness did not have any added value. Given that visual MTA rating, which is easier to obtain in clinical settings, performed almost as well as brain volumes, it may represent a useful alternative to the more complex volumetric measures.

Apolipoprotein E ε4 carrier status was very effective in improving the results and adding *APOE* to basic clinical data was almost as effective as performing an MRI scan. *APOE* and MRI together, in the absence of any other factors, led to better performance compared with the complete model (AUC 0.81–0.82 vs. 0.78). This is because the model showed that several factors in the demographic, cardiovascular and cognitive groups were not useful in detecting amyloid positivity.

Regarding cognition, executive functioning was most effective, with an individual AUC of about the same magnitude as *APOE* genotype. Cognition as a group was, however, not as valuable in different combinations of factor modalities as *APOE* or MRI measures. In contrast, the previous SVM model (ten Kate et al., 2018) emphasized memory among the tested cognitive domains. This may be due to population differences, i.e., FINGER participants underwent cognitive screening to select individuals with performance at the mean level or slightly lower than expected for age, thus limiting the distribution of cognitive test scores in the present study.

Among vascular factors, BMI had some ability to detect amyloid positivity, but not hypertension. Low BMI at younger-old ages has previously been associated with Aβ load (Ewers et al., 2012; Toledo et al., 2012), although these studies also included individuals with MCI and dementia at baseline. However, the performance of vascular factors may have been influenced by the use of the CAIDE dementia risk score (including age, sex, education, BMI, systolic blood pressure, total serum cholesterol and physical activity) (Kivipelto et al., 2006) to select the at-risk FINGER study participants. FINGER eligibility criteria may also explain why age did not associate with Aβ positivity in this population, despite being reported as a clear determinant of Aβ pathology in individuals with normal cognition or MCI, with Aβ pathology prevalence growing rapidly after about the age of 70 years (Jansen et al., 2015).

The main limitations of the present study are the small sample size leading to potential model overfitting effects, and the lack of external validation. However, while the sample size is limited, the ratio of amyloid positive and negative cases is balanced. The same dataset was used for both training and testing the DSI model, although we reported results following nested 100 × 5 cross-validation. Findings need to be interpreted keeping in mind that FINGER participants had already undergone a screening process based on cognitive testing and the CAIDE dementia risk score, i.e., they represent a population at risk for dementia, but without dementia or substantial cognitive impairment. Studies in independent populations will be needed to further validate the results. Testing the model with a larger sample could show more effects for age, cognition and vascular factors. There was no significant association between age and amyloid positivity in our sample, likely due to the original selection process, which only recruited participants aged 60–77, who were at-risk for cognitive impairment. This would exclude healthier young subjects and more cognitively impaired older subjects, which could make the amyloid-age association less prominent.

Compared with previous studies in cognitively normal populations, the present study assessed a broader range of factors, and performance at multiple levels simultaneously, i.e., from the overall model to groups of conceptually related factors and also individual factors. We also investigated different screening strategies, i.e., the benefit of adding more complex factor modalities, by testing the performance of increasingly comprehensive models, from easily obtainable demographic, clinical and cognitive data, to *APOE* genotyping and structural brain MRI.

## Data Availability Statement

The datasets for this article are not publicly available because of privacy issues of sensitive personal data. Requests to access the datasets should be directed to AS, alina.solomon@uef.fi.

## Ethics Statement

The studies involving human participants were reviewed and approved by Coordinating Ethics Committee of the Hospital District of Helsinki and Uusimaa. The patients or participants provided their written informed consent to participate in this study.

## Author Contributions

TPe, AH, TN, JL, JR, HS, MK, and AS: designing the study. TN, SH, TH, NK, YL, TPa, JR, HS, and MK: acquisition of the data. TPe, AH, TN, MG, JL, NK, JR, and AS: interpretation of the data. TPe: analysis of the data. TPe, AH, and AS: drafting the manuscript. All authors contributed in revising the manuscript, read and approved the final version.

## Conflict of Interest

JL was shareholder and co-founder of Combinostics Ltd. Combinostics Ltd., owns the following IPR related to the manuscript: (1) A method for inferring the state of a system, US7, 840,510 B2, and PCT/FI2007/050277. (2) State Inference in a heterogeneous system, PCT/FI2010/050545 and FI20125177. The remaining authors declare that the research was conducted in the absence of any commercial or financial relationships that could be construed as a potential conflict of interest.

## Footnotes

1 http://surfer.nmr.mgh.harvard.edu
